# Supplementary material for: Validating TDP1 as an Inhibition Target for Lipophilic Nucleoside Derivative in Human Cells
Source: Int J Mol Sci. 2025 Oct 20;26(20):10193. doi: 10.3390/ijms262010193 (PMC12565254; doi:10.3390/ijms262010193)
Supplement: Supplementary file 1 [file ijms-26-10193-s001.zip › ijms-3913418-supplementary.pdf]

Supplementary materials

Validating TDP1 as an Inhibition Target for Lipophilic Nucleoside Derivative in Human Cells

Irina A. Chernyshova [1], Tatyana E. Kornienko [1], Nadezhda S. Dyrkheeva [1], Alexandra L. Zakharenko [1], Arina A. Chepanova [1], Konstantin E. Orishchenko [2], Nikolay N. Kurochkin [3], Mikhail S. Drenichev [3] and Olga I. Lavrik [1,*]

[1] Institute of Chemical Biology and Fundamental Medicine, Siberian Branch of the Russian Academy of Sciences, 630090 Novosibirsk, Russia; chernyshova0305@gmail.com (I.A.C.); t.kornienko1995@gmail.com (T.E.K.); dyrkheeva.n.s@gmail.com (N.S.D.); a.zakharenko73@gmail.com (A.L.Z.); arinachepanova@mail.ru (A.A.C.)
[2] Federal Research Centre Institute of Cytology and Genetics, Siberian Branch of the Russian Academy of Sciences, 630090 Novosibirsk, Russia; orishchenkoke@icg.sbras.ru
[3] Engelhardt Institute of Molecular Biology, Russian Academy of Sciences, 119991 Moscow, Russia; nikola.76@mail.ru (N.N.K.); mdrenichev@mail.ru (M.S.D.)
* Correspondence: lavrik@1bio.ru.

Table S1. Combination indexes (CI) for the combination of compound **6d** and Tpc, calculated using the MTT test on A549WT cells.

Dose Tpc	Dose 6d	Effect	CI	Dose Tpc	Dose 6d	Effect	CI
3.0	5.0	0.05	6.28621	3.0	10.0	0.07	3.91290
8.0	5.0	0.01	795.098	8.0	10.0	0.08	5.73736
20.0	5.0	0.15	2.42159	20.0	10.0	0.16	2.65377
51.0	5.0	0.28	1.09167	51.0	10.0	0.31	1.24913
128.0	5.0	0.45	0.57309	128.0	10.0	0.5	0.71007
320.0	5.0	0.48	0.79742	320.0	10.0	0.55	0.75455
800.0	5.0	0.55	0.89911	800.0	10.0	0.64	0.67916
2000.0	5.0	0.65	0.79212	2000.0	10.0	0.74	0.54600
5000.0	5.0	0.77	0.50595	5000.0	10.0	0.87	0.29590

A549	WT	B5	B6	B10
CC_{50} Tpc, nM	457±29	152±14	28±3	163±27

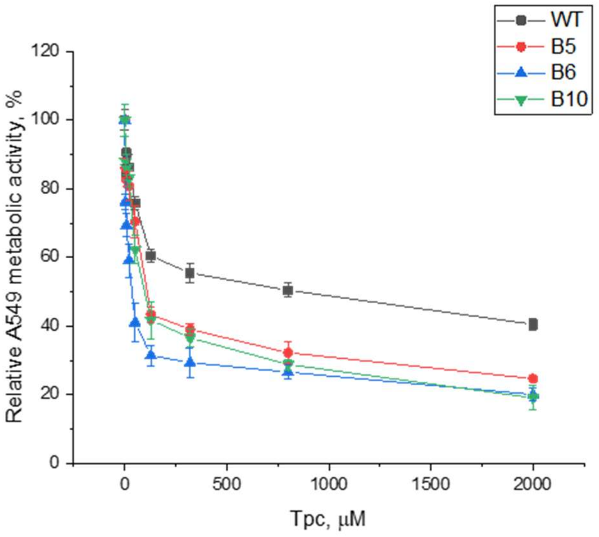

Figure S1. The relative A549 WT and TDP1-KO (cell clones B5, B6, B10) metabolic activity curves in presence of topotecan (Tpc). The data were obtained by the MTT test.

A549	WT	B5	B6	B10
CC_{50} 6d, μM	22±1	53±2	43±2	42±3

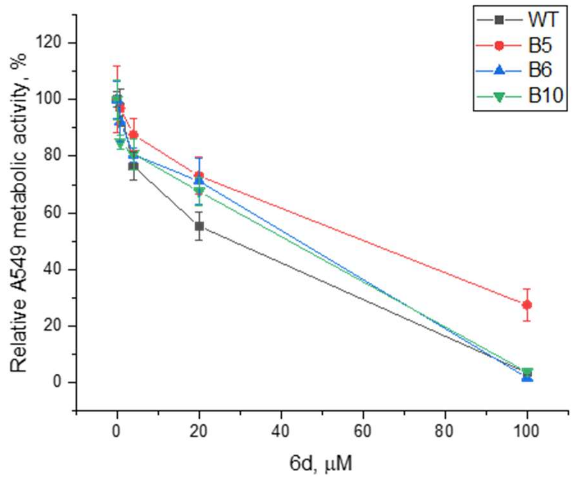

Figure S2. The relative A549 WT and TDP1-KO (cell clones B5, B6, B10) metabolic activity curves in presence of compound **6d**. The data were obtained by the MTT test.

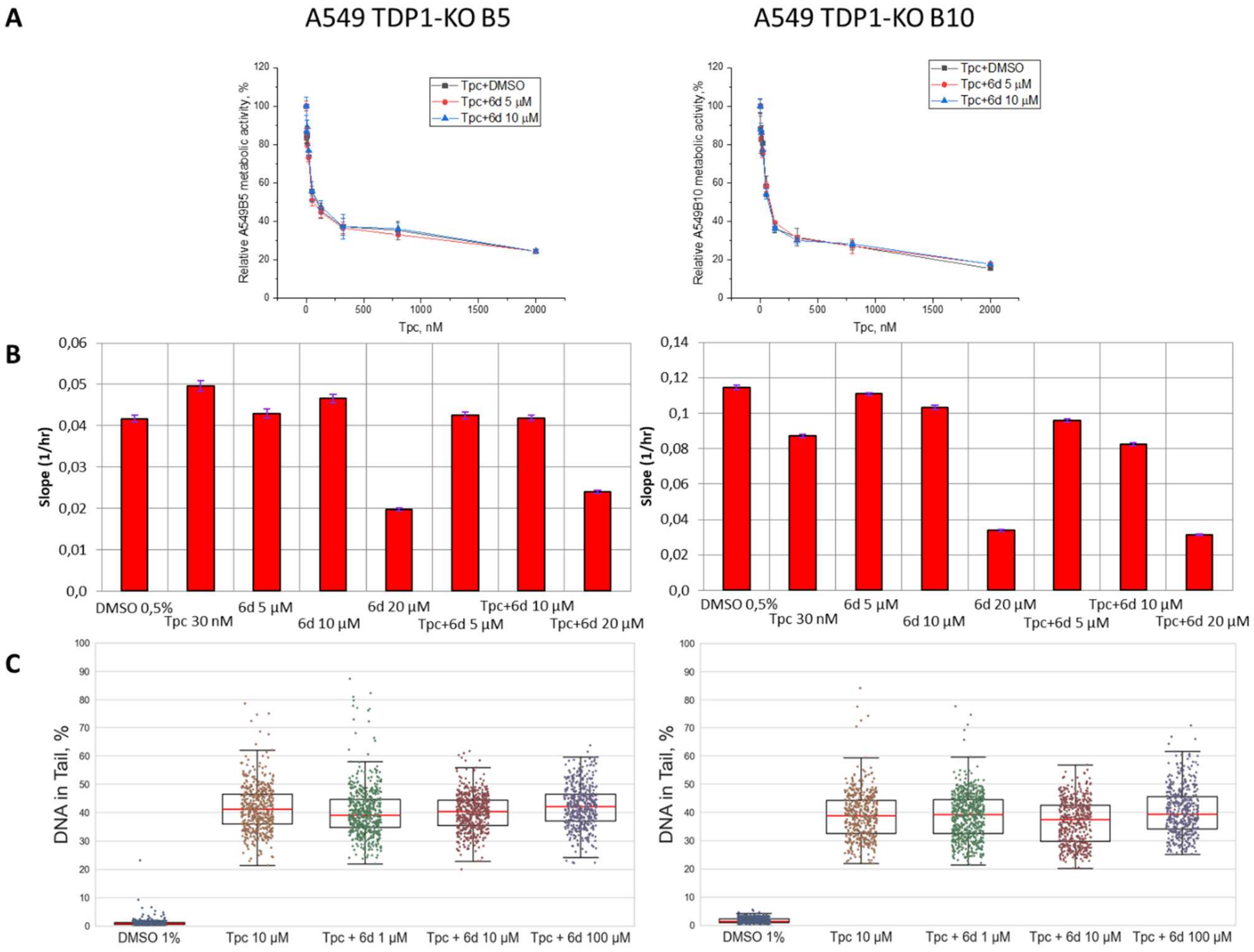

Figure S3. Study of the effect of compound **6d** on the action of Tpc in A549 TDP1-KO B5 and B10 cells. **A)** The relative cell metabolic activity curves were obtained by the MTT assay. Combined treatment with Tpc and **6d** does not result in decreased relative metabolic activity compared to Tpc alone in either cell line. **B)** Cell indexes data were obtained by impedance-based real-time assay on the xCELLigence System. Combined treatment with Tpc and **6d** does not decrease cell index compared to individual Tpc treatment in B5 (**left**) and B6 (**right**) cells. **C)** Level of cellular DNA damage assessed by Alkaline Comet assay. The percentage of DNA in the tail for TDP1-KO B5 (**left**) and B10 (**right**) cells in the groups with Tpc and combination treatments does not statistically differ (Tukey's HSD).

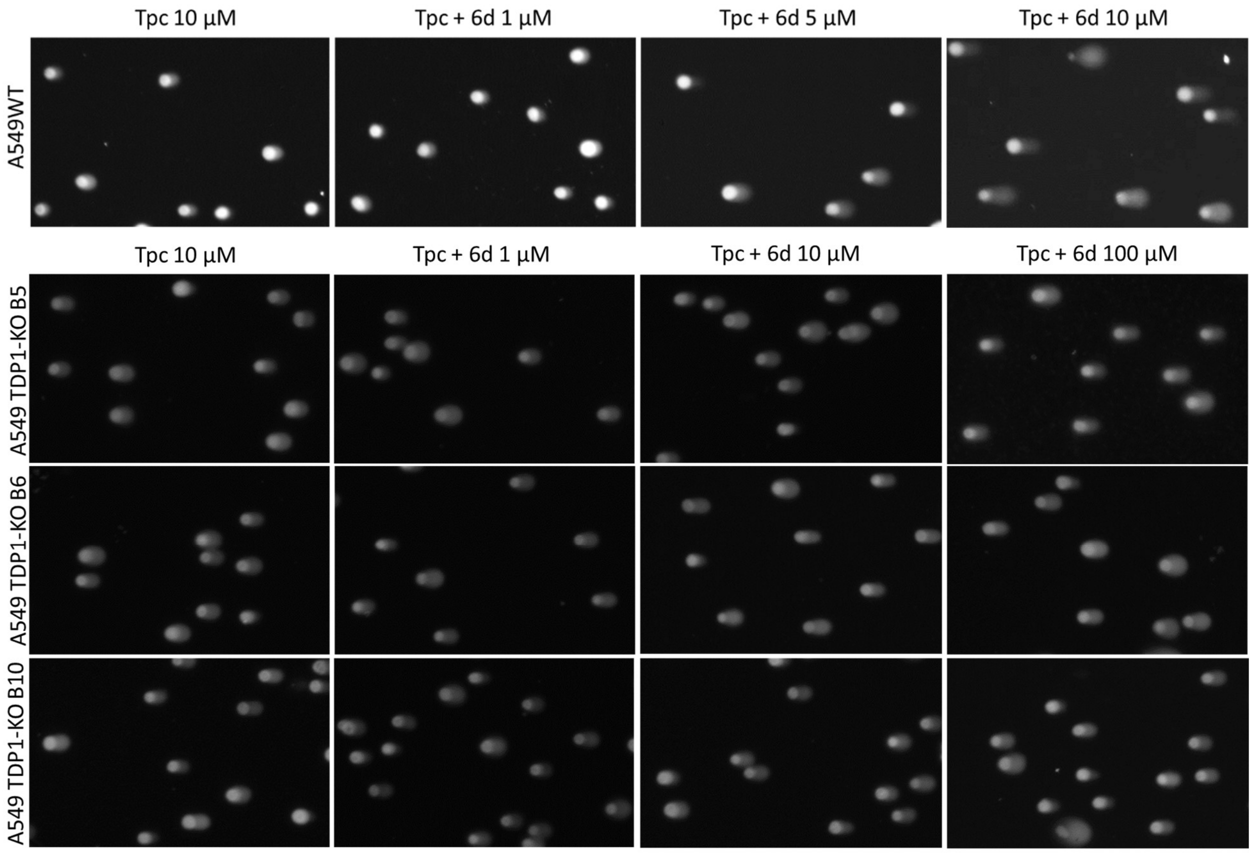

Figure S4. Microphotographs of the slides were obtained by Alkaline Comet assay. For A549 WT cells, an increase in % DNA in the tail is characteristic of the groups treated with the combination of compound **6d** and Tpc compared to the Tpc group. For A549 TDP1-KO cells, "comets" appear identical and percentage of the DNA in the tail remains constant across all treatment types.

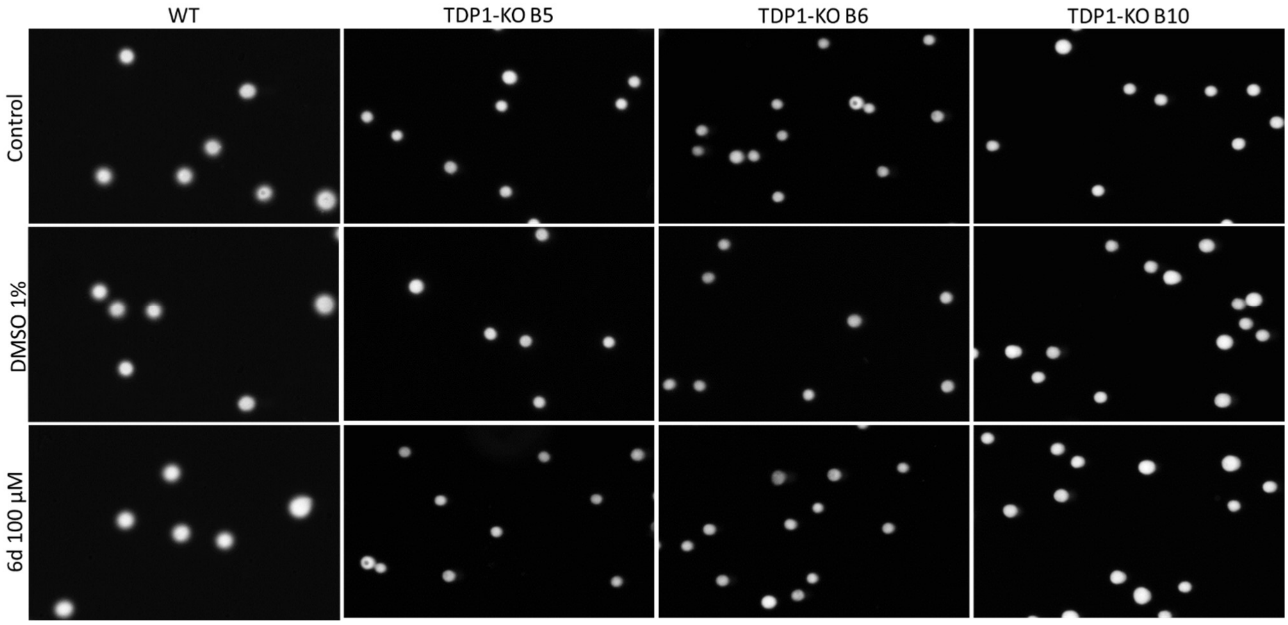

Figure S5. Microphotographs of slides were obtained by Alkaline Comet assay. Control A549WT and TDP1-KO cells, solvent control and compound **6d** are identical and do not show DNA damage.

HEK293A	WT	C6	G6	F7
CC_{50} 6d, µM	22±1	26±1	20±2	26±3

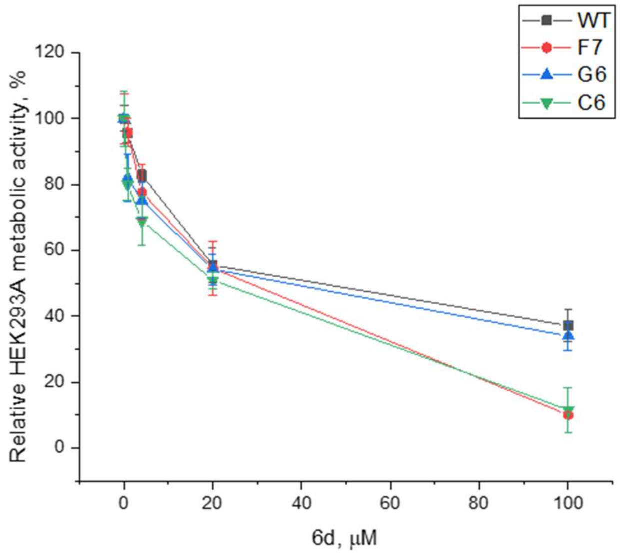

Figure S6. The relative HEK293A WT and TDP1-KO (cell clones C6, G6, F7) metabolic activity curves in presence of compound **6d**. The data were obtained by the MTT test.

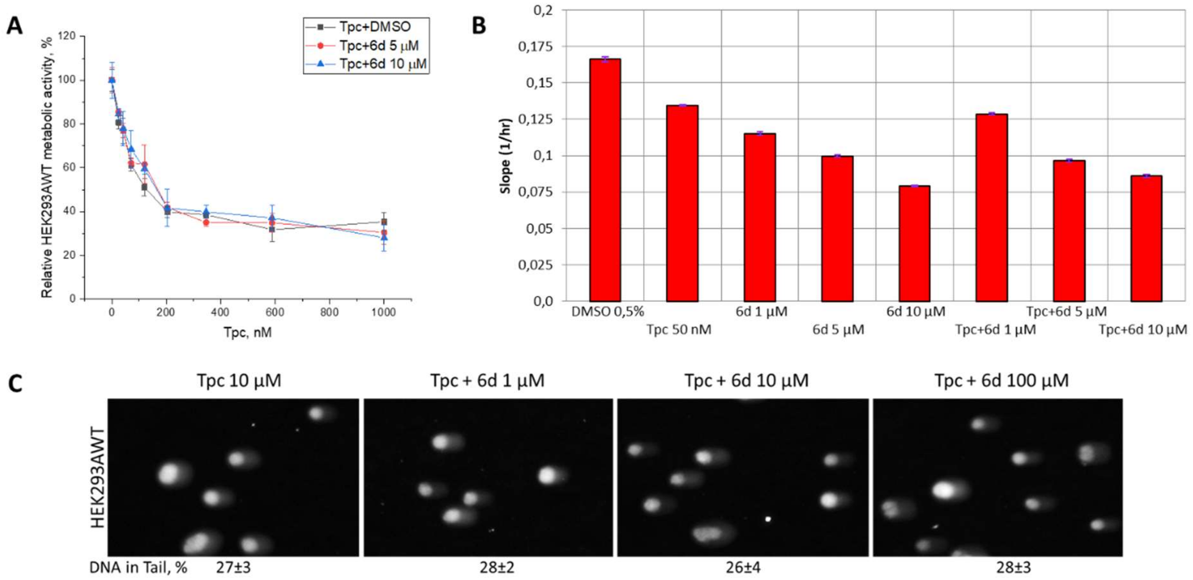

Figure S7. The effect of compound **6d** on Tpc in HEK293A WT cells. **A)** The relative cell metabolic activity curves were obtained by MTT, **B)** cell indexes were obtained by impedance-based real-time assay, **C)** Comet assay images. DNA damage in HEK293A WT with Tpc ± **6d** was the same (tail lengths and % DNA in tail were similar across groups).

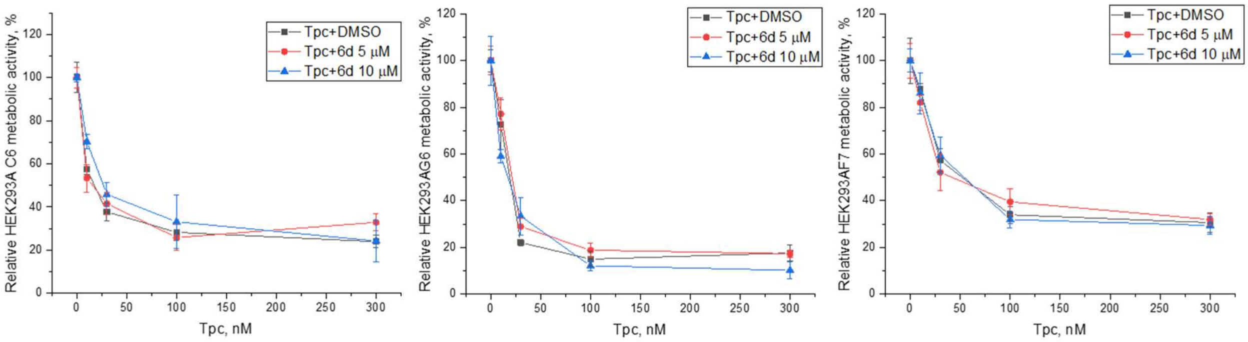

Figure S8. The relative HEK293A TDP1-KO (cell clones C6, G6, F7) metabolic activity curves under Tpc in the presence of compound **6d** at a non-toxic concentration. The data were obtained by the MTT test.

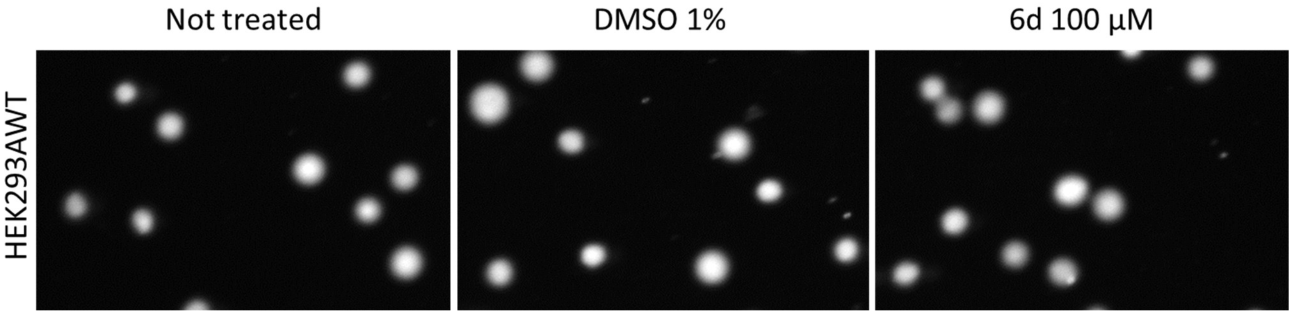

Figure S9. Microphotographs of slides were obtained by Alkaline Comet assay. Control HEK293AWT cells, solvent control and compound **6d** are identical and do not show DNA damage.

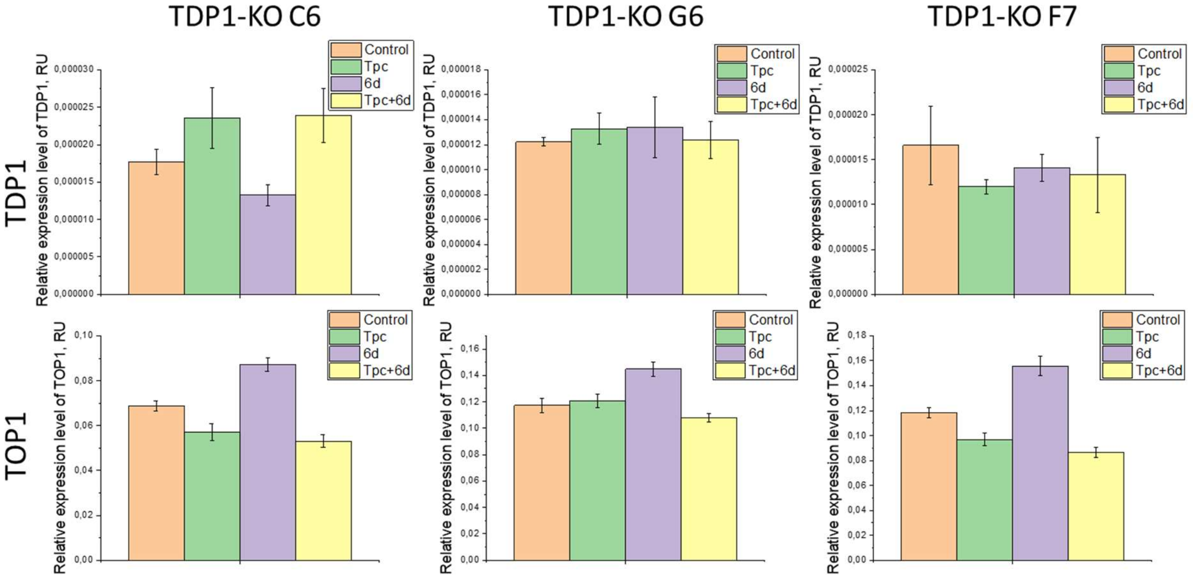

Figure S10. Relative expression levels of TDP1 and TOP1 in HEK293A TDP1-KO (C5, C6, F7) cells under Tpc, compound **6d** and their combination. All types of treatment did not differ significantly compared to the control group (Tukey's HSD).

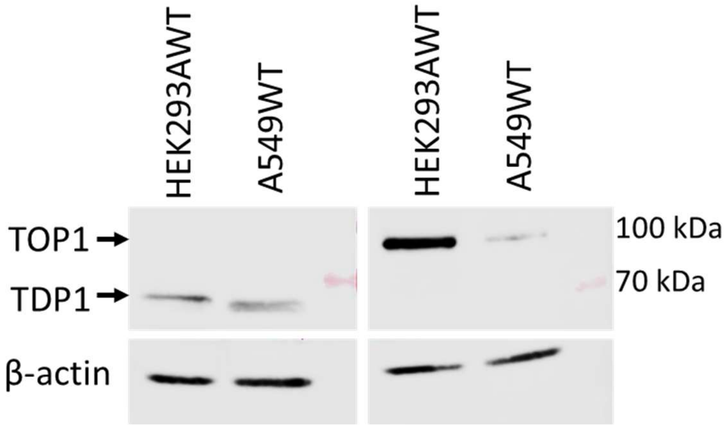

Figure S11. Western blot analysis of whole-cell extracts from HEK293A and A549 wild-type cells. The TDP1 content was similar in both cell types, while the TOP1 content in non-tumor HEK293A cells was significantly higher than in tumor A549 cells.
